# Identification of four *TMC1* variations in different Chinese families with hereditary hearing loss

**DOI:** 10.1002/mgg3.394

**Published:** 2018-04-14

**Authors:** Hongyang Wang, Kaiwen Wu, Jing Guan, Ju Yang, Linyi Xie, Fen Xiong, Lan Lan, Dayong Wang, Qiuju Wang

**Affiliations:** ^1^ Institute of Otolaryngology Chinese PLA General Hospital Medical School of Chinese PLA Beijing China

**Keywords:** hereditary hearing loss, high‐throughput sequencing, *TMC1*, variant

## Abstract

**Background:**

Variants in *TMC1* (transmembrane channel‐like 1) can cause both autosomal dominant and recessive hearing loss in human population. Mice with *Tmc1* variants have been shown to be ideal animal models for gene therapy. In this article, we report four *TMC1* variants in four different Chinese families and the follow‐up auditory phenotype of a previously reported family.

**Methods:**

Four families with *TMC1* variants, as well as a previously described family with *TMC1* variant orthologous to the Beethoven mouse, were recruited in this study. A comprehensive auditory evaluation was performed on all ascertained family members. High‐throughput sequencing was conducted using genomic DNA from the probands and other family members to identify probable deafness genes.

**Results:**

We identified four *TMC1* (NM_138691.2) variations, including two pathogenic variants, c.1714G>A, and c.1253T>A, one likely pathogenic variant, c.[797T>C];[797T>C], and one single nucleotide polymorphism (SNP), c.2276G>A. Among these variants, c.[797T>C];[797T>C] is a novel likely pathogenic variant, and c.1714G>A and c.1253T>A are known pathogenic variants at the DFNB7/11 (DFNA36) locus. Phenotype‐genotype correlation analysis of *TMC1* variants showed that the *TMC1* dominant variation‐related phenotype was late‐onset, progressive, high frequency to all frequency sensorineural hearing loss, while the *TMC1* recessive variant was related to congenital all frequency sensorineural hearing impairment.

**Conclusions:**

Two pathogenic, one likely pathogenic variants and one SNP of *TMC1* were identified in four Chinese families with hereditary hearing loss, indicating that *TMC1* may be a more frequent cause of hearing loss than expected. *TMC1* variants related to hearing loss result in specific phenotypes. The *TMC1* c.1253T>A (p.M418K) variation, homologous to the *Tmc1* c. 1235 T> A (p.M412K) variant in Beethoven mice, was the second report of this variant in human patients with hearing loss, suggesting the possibility to translational gene therapy from Beethoven mice to human patients.

## 1 INTRODUCTION

Hearing loss is one of the most common sensorineural defects in humans worldwide. The prevalence is 1/1,000 in newborns and increases to 2.7/1,000 before 5 years old and to 3.5/1,000 during adolescence (Morton & Nance, 2006). At least 50% of hearing impairment cases are caused by genetic factors and are classified as syndromic and nonsyndromic hearing loss, which can be subclassified to autosomal recessive nonsyndromic hearing loss (ARNSHL, accounts for 77%), autosomal dominant nonsyndromic hearing loss (ADNSHL, 22%), X‐linked hearing loss, Y‐linked hearing loss, and mitochondrial hearing loss. Hereditary hearing loss is a typical monogenic disorder with high heterogeneity, and at least 141 genes have been reported thus far (http://hereditaryhearingloss.org/ 2017‐10‐5), among which eight genes, including *COL11A2, GJB2, GJB6, MYO6, MYO7A, TBC1D24, TECTA,* and *TMC1,* can cause both ARNSHL and ADNSHL.

**Figure 1:**
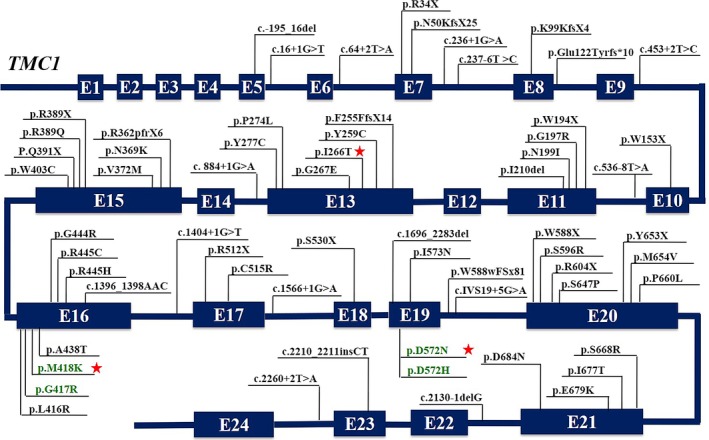
Variant spectrum of *TMC1*. The mode figure of *TMC1* shows 24 exons and introns. The black words and green words indicate variants associated with DFNB7/11 and DFNA36 hearing loss respectively. Three red stars indicate variants of *TMC1* in this study

The transmembrane channel‐like 1 (*TMC1*, OMIM: 606706) gene is the sixth most frequent cause of hereditary hearing loss among the ARNSHL‐related genes based on worldwide case studies, following *GJB2, SLC26A4, MYO15A, OTOF,* and *CDH23* (Hilgert, Smith, & Van Camp, 2009). The prevalence of *TMC1* variants was 3.4% (19/557) among Pakistani ARNSHL families (Kitajiri, McNamara, et al., 2007), 4.3% (4/93)(Kalay et al., 2005) to 8.1% (7/86)(Sirmaci et al., 2009) in Turkish families, 5.9% (5/85) in Tunisia (Tlili et al., 2008), and 4.2% (1/24) in Europe (Schrauwen et al., 2013). *TMC1* variants at the DFNB7/11 and DFNA36 locus are implicated in the pathogenesis of both ARNSHL and ADNSHL respectively. The former appears primarily as congenital or prelingual severe/profound hearing impairment, while the latter shows postlingual progressively aggravated hearing loss. To date, 59 different types of DFNB7/11 variants and four DFNA36 variants have been identified in hereditary hearing loss families (Figure 1 and Appendix S1; Bademci et al., 2016; Bakhchane et al., 2015; Brownstein et al., 2011; Chen et al., 2015; Davoudi‐Dehaghani, Fallah, Tavakkoly‐Bazzaz, Bagherian, & Zeinali, 2015; de Heer et al., 2011; Ganapathy et al., 2014; Gao et al., 2015, 2013; Hilgert et al., 2008; Hu et al., 2016; Imtiaz et al., 2016; Kalay et al., 2005; Kitajiri, Makishima, Friedman, & Griffith, 2007; Kitajiri, McNamara, et al., 2007; Kurima et al., 2002; Lin et al., 2014; Meyer et al., 2005; Riahi et al., 2014; Santos et al., 2005; Schrauwen et al., 2013; Shafique et al., 2014; Sirmaci et al., 2009; Tlili et al., 2008; Yang et al., 2010; Yang, Wei, Chai, Li, & Wu, 2013; Zhao et al., 2014). With the rapid development in sequencing technology, such as high‐throughput sequencing (HTS), which has the advantages of low sample quantity, low cost, and high efficiency, more *TMC1* variants have been reported in various regions, such as Ecuador, Algerian, Morocco, and China.

The Beethoven (*Bth*) mouse is a natural model with hearing impairment that carries the *Tmc1* (NM_028953.2) missense variant (p.M412K, c.T1235A), which is orthologous to that found in Family 1304, a DFNA36 family we previously described (Zhao et al., 2014). This dominant variant can cause progressive and profound hearing loss in humans. Recently, gene therapy in the *Bth* mouse model by delivery of genome editing agents was shown to be effective (Xue Gao et al., 2017). The successful treatment of the *Bth* mouse suggests possible strategies to correct *TMC1* variants and potentially rescue hearing in human patients.

In this study, we identified four variants in *TMC1*, including a novel likely pathogenic recessive variant, c.[797T>C];[797T>C] (p.[I266T];[=]); two known dominant variants, c.1714G>A (p.D572N) and c.1253T>A (p.M418K); and a single nucleotide polymorphism (SNP), c.2276G>A (p.R759H), from four different Chinese families by HTS. The two pathogenic dominant variants all cosegregated with hearing loss in the families, and the recessive variant is verified to be likely pathogenic for now. All of them were not present in 1700 Chinese controls with normal hearing. In addition, we performed audiology follow‐up in Family 1304 (c.1253T>A p.M418K) to evaluate the progression of hearing loss (Zhao et al., 2014).

## 2 METHODS AND MATERIALS

### 2.1 Ethical compliance

The study was approved by the Committee of Medical Ethics of the Chinese PLA General Hospital. Written informed consent was collected from all participants in the five families.

### 2.2 Family recruitment and clinical evaluations

Family 01 is a four‐generation family from Shanxi Province, with 12 individuals ascertained in this study, including five affected patients and seven interfamilial normal members. Family 02 is a four‐generation family with five affected members from the Shandong Province. Family 03 is a small hearing loss family with three members, including a patient and her parents, who had a normal hearing. Family 04 is a five‐generation family with 11 affected members from Inner Mongolia. Family 1304 is a large ADNSHL family that was previously described in 2014 (Zhao et al., 2014); some of the affected members from Family 1304 were followed up in 2015. All participants in this study were ethnic Han. The five families were ascertained at the Institute of Otolaryngology, Chinese PLA General Hospital. Medical histories were obtained using a questionnaire, and general physical examinations investigating hearing loss, tinnitus, vestibular symptoms, and other clinical abnormalities were performed by experienced physicians and audiologists to exclude syndromic hearing loss. Audiometric evaluations included pure tone audiometry (PTA), acoustic immittance measurement, distortion product otoacoustic emissions (DPOAE), auditory brainstem responses (ABR), 40 Hz auditory event related potential (AERP), and speech recognition. PTA was calculated as the average of the hearing threshold of patients at 500, 1,000, 2,000, and 4,000 Hz. The degree of hearing loss was defined as mild (26–40 dB HL), moderate (41–55 dB HL), moderately severe (56–70 dB HL), severe (71–90 dB HL), and profound (>90 dB HL). High resolution computed tomography (HRCT) was also performed on the probands to exclude abnormal structure of middle and inner ears.

### 2.3 Sanger sequencing

The genomic DNA (gDNA) of peripheral blood was extracted from all participants in the four families using a Blood DNA kit according to the standard protocol (TIANGEN BIOTECH, Beijing, China). Polymerase chain reaction (PCR) and Sanger sequencing were performed on the propositus and available members to determine whether the potential variant cosegregated with the disease phenotype in the family. Direct PCR products were sequenced using BigDye terminator v3.1 cycle sequencing kits (Applied Biosystems. Foster City, CA) and analyzed using an ABI 3700XL Genetic Analyzer. The primer sequences and PCR cycle used are provided in Appendix S2. We also selected 1,700 blood samples from healthy Chinese individuals with normal hearing as controls.

### 2.4 Targeted gene capture and HTS

One microgram of gDNA from peripheral blood samples was fragmented to 200–300 base pairs using an ultrasonoscope (Covaris S2, Massachusetts, USA). End‐repair, adenylation and adapter ligation were performed for library preparation following Illumina's protocol (Wang et al., 2014). The same amount of the library was pooled and then hybridized to the customized capture array (NimbleGen, Roche), which had exons, splicing sites and immediate flanking intron sequences of 127 genes (Appendix S3), including most of the known genes involved in nonsyndromic hearing loss and those involved in some relatively common syndromic hearing loss conditions. Sequencing was carried out on an Illumina HiSeq2000 to generate paired end reads (90 bps at each end). Reads were aligned to the NCBI37/hg19 assembly using the BWA (Burrows Wheeler Aligner). SNPs and indels (inserts and deletions) were detected using GATK software. Potentially pathogenic variants were defined as nonsense, missense, splice‐site, and indel variants that had allele frequencies under 0.01 (determined by the NCBI dbSNP, NHLBI ESP and 1000Genomes databases). Possible pathogenic effects of the missense variants were evaluated by computational tools, including MutationTaster, PolyPhen‐2, and SIFT.

## 3 RESULTS

### 3.1 Clinical description

#### 3.1.1 Family 01

**Figure 2:**
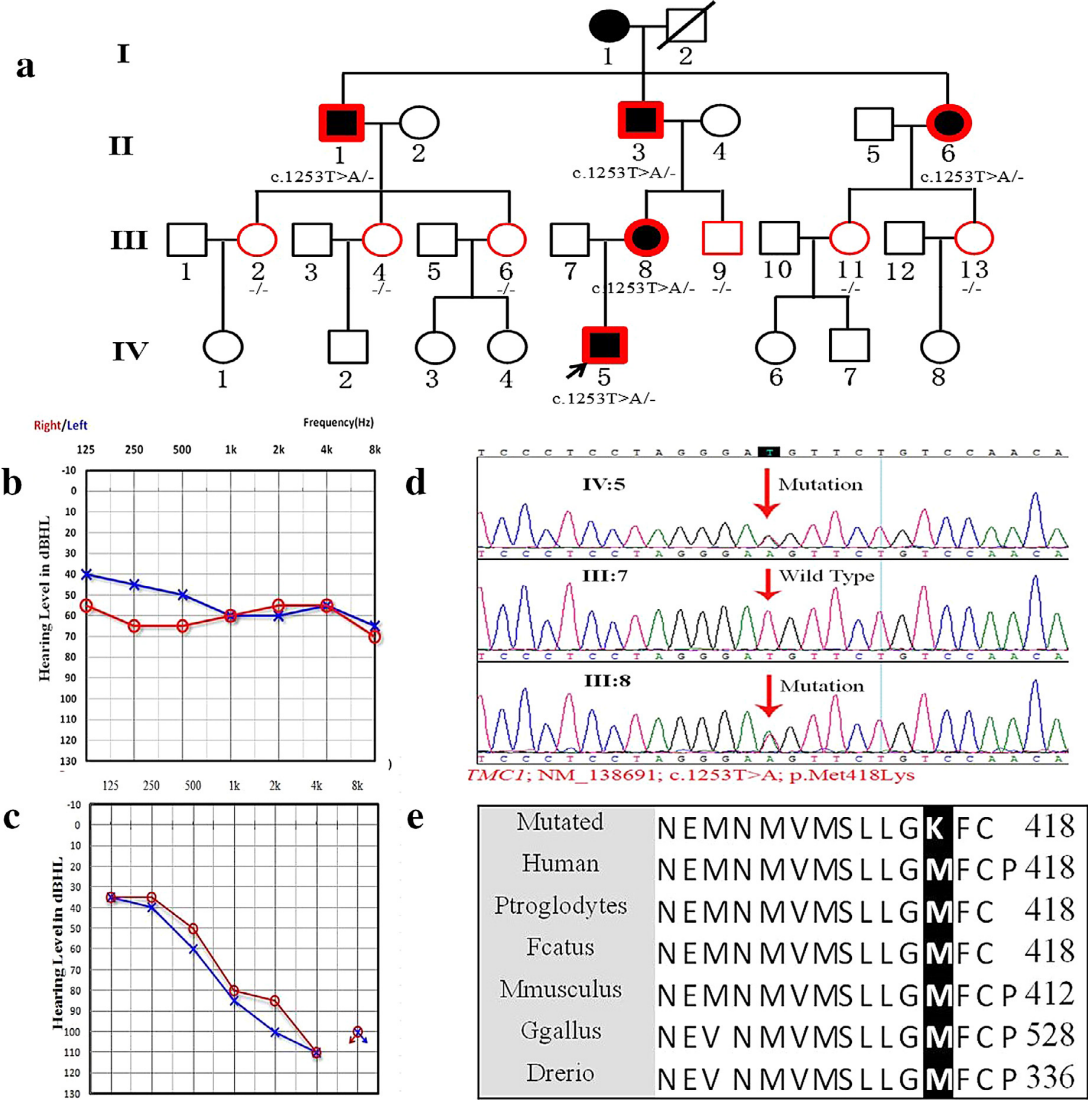
Genotype and phenotype of Family 01. (a) Pedigree of Chinese Family 01 with ADNSHL. Affected subjects are denoted in black. The proband is indicated by an arrow. Symbols with red frame indicate members who have been sequenced in this study, and *TMC1* gene testing results are listed below the symbols. (b) Audiogram of the proband. (c) Audiogram of the proband's mother, who is 32 years old now. (d) Sequencing chromatograms of *TMC1* show the variant c.1253T>C (p.M418K) in the proband and other members of the family. (e) The methionine, substituted by lysine at position 418 of *TMC1*, is evolutionarily conserved among different species

Family 01 was a four‐generation family suffering from ADNSHL (Figure 2). The propositus was a 9‐year‐old boy, who displayed hearing loss at 8 years of age. He had a history of gentamicin injection several years earlier. The PTA was 56 dB HL and 61 dB HL in the left and right ear respectively. The threshold of ABR was 80 dB nHL, and the latencies of wave V were prolonged in both ears. The DPOAE could be evoked at all frequencies. No other obvious systemic features were identified by the medical history and physical examination. His mother, a 32‐year‐old woman, complained of tinnitus at 16 years old and hearing loss at approximately 20 years old, displaying all frequency hearing impairment, with no symptoms of vestibular dysfunction.

#### 3.1.2 Family 02

**Figure 3:**
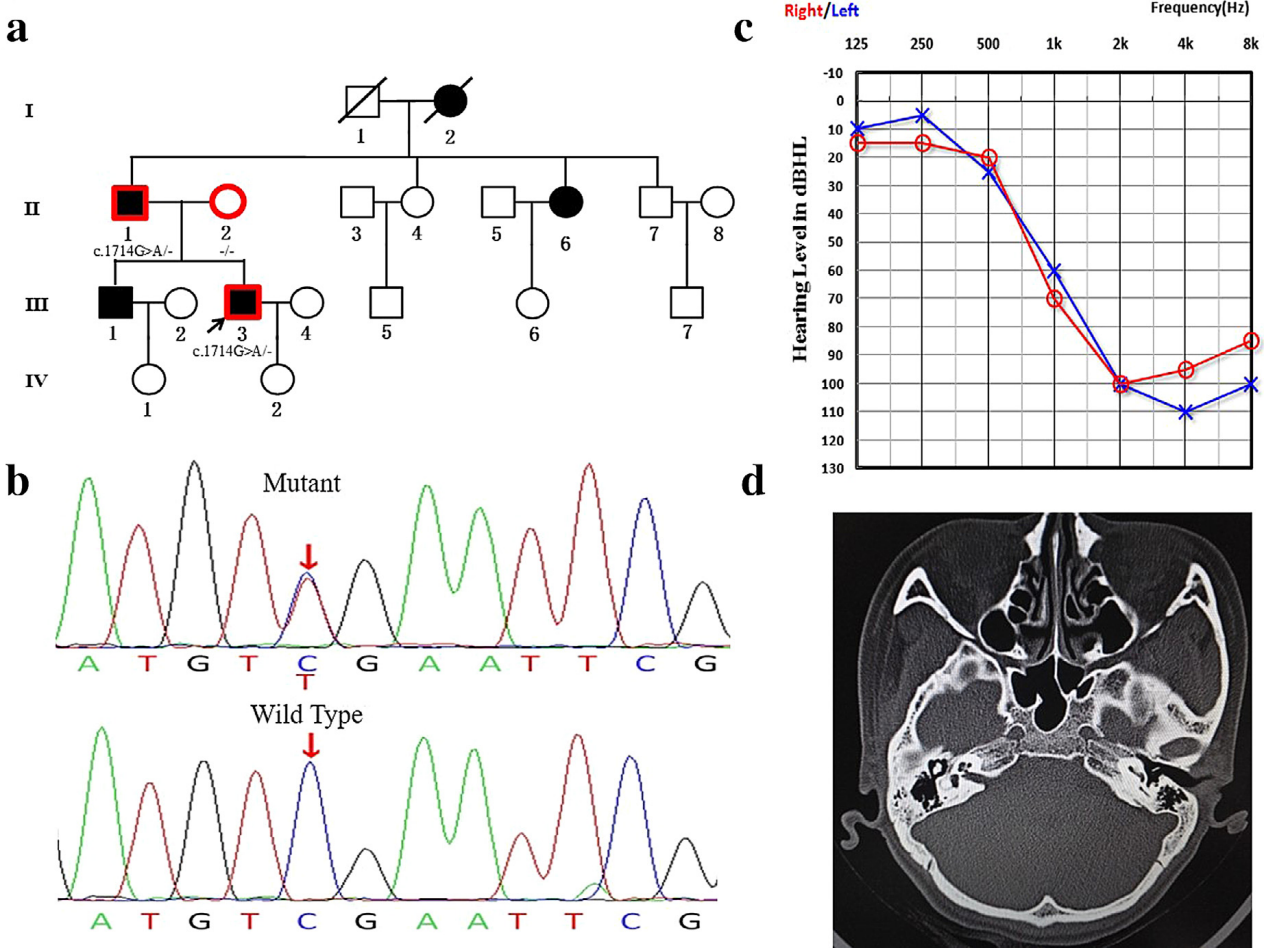
Genotype and phenotype of Family 02. (a) Pedigree of Chinese Family 02 with ADNSHL. Affected subjects are denoted in black. The proband is indicated by an arrow. Symbols with red frame indicate members who have been sequenced in this study, and *TMC1* gene testing results are listed below the symbols. (b) Sequencing chromatograms (antisense sequence) of *TMC1* show the variant c.1714G>A in affected and wild type in normal individuals. (c) Audiogram of the proband. (d) HRCT of temporal bone of the proband

Family 02 was a four‐generation pedigree with 19 members, including five affected and 15 unaffected individuals (Figure 3). The affected members showed postlingual, progressive and high‐frequency sensorineural hearing impairment. The propositus (III:3) was a 36‐year‐old male when he first visited the out‐patient clinic in the year 2016. He suffered high‐frequency hearing impairment accompanied by tinnitus. Tympanogram showed a type A curve, indicating normal functions of both middle ears. Speech recognition was 40% and 48% in the left and right ears, respectively. HRCT of temporal bone indicated normal middle and inner ear structure. No other special abnormal finding was discovered by the medical history and physical examination.

#### 3.1.3 Family 03

**Figure 4:**
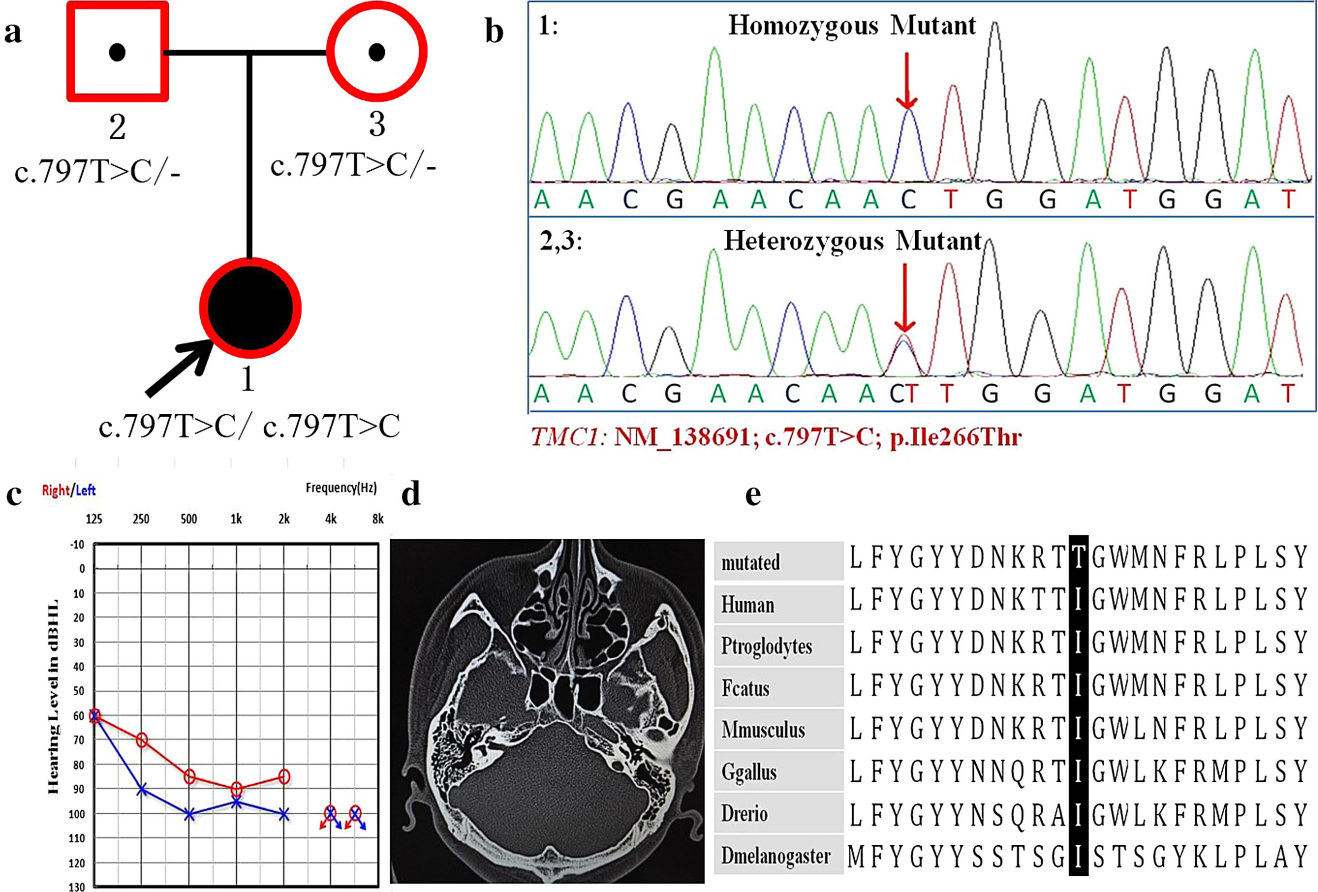
Genotype and phenotype of Family 03. (a) Pedigree of Chinese Family 03 with ARNSHL. Symbols with red frame indicate members who have been sequenced in this study, and *TMC1* gene testing results are listed below in the symbols. (b) Sequencing chromatograms of *TMC1* show the homozygous variant c.[797T>C];[797T>C] in the proband and the heterozygous variant in her parents. (c) Audiogram of the proband. (d) HRCT of temporal bone of the proband. (e) The isoleucine, substituted by tyrosine at position 266 of *TMC1*, is evolutionarily conserved among different species

Family 03 was a small pedigree with 3 members who suffered from ARNSHL (Figure 4). The propositus was a 26‐year‐old female, who suffered from pre‐lingual severe to profound hearing loss. Tympanogram showed a type A curve. The ABR could not be elicited, and the DPOAEs were absent at all frequencies in both ears. HRCT of temporal bone scans indicated normal middle and inner ear structure. Professional physical examination and medical history were performed to exclude the possibility of common syndromic deafness.

#### 3.1.4 Family 04 (Figure S1)

Family 04 was a five‐generation pedigree affected by ADNSHL with 10 affected members. The propositus (V:8) displayed hearing loss when he was 2 years old. The threshold of ABR was 65 dB nHL, the 40 Hz AERP was 70 dB nHL and DPOAEs were not evoked at all frequencies for both ears. The features of affected adults with hearing impairment were similar to those of Family 01. The age of onset ranged from 2 (V:8) to 25 (III:12) years old, and severity of hearing loss was aggravated with the individual's age. The low‐frequency hearing threshold was initially in a normal range; however, all frequencies deteriorated by the fifth decade. Specific histories, such as exposure to noise or ototoxic drugs, were excluded, and no other related systemic features were identified by the medical history and physical examination.

#### 3.1.5 Family 1304

**Figure 5:**
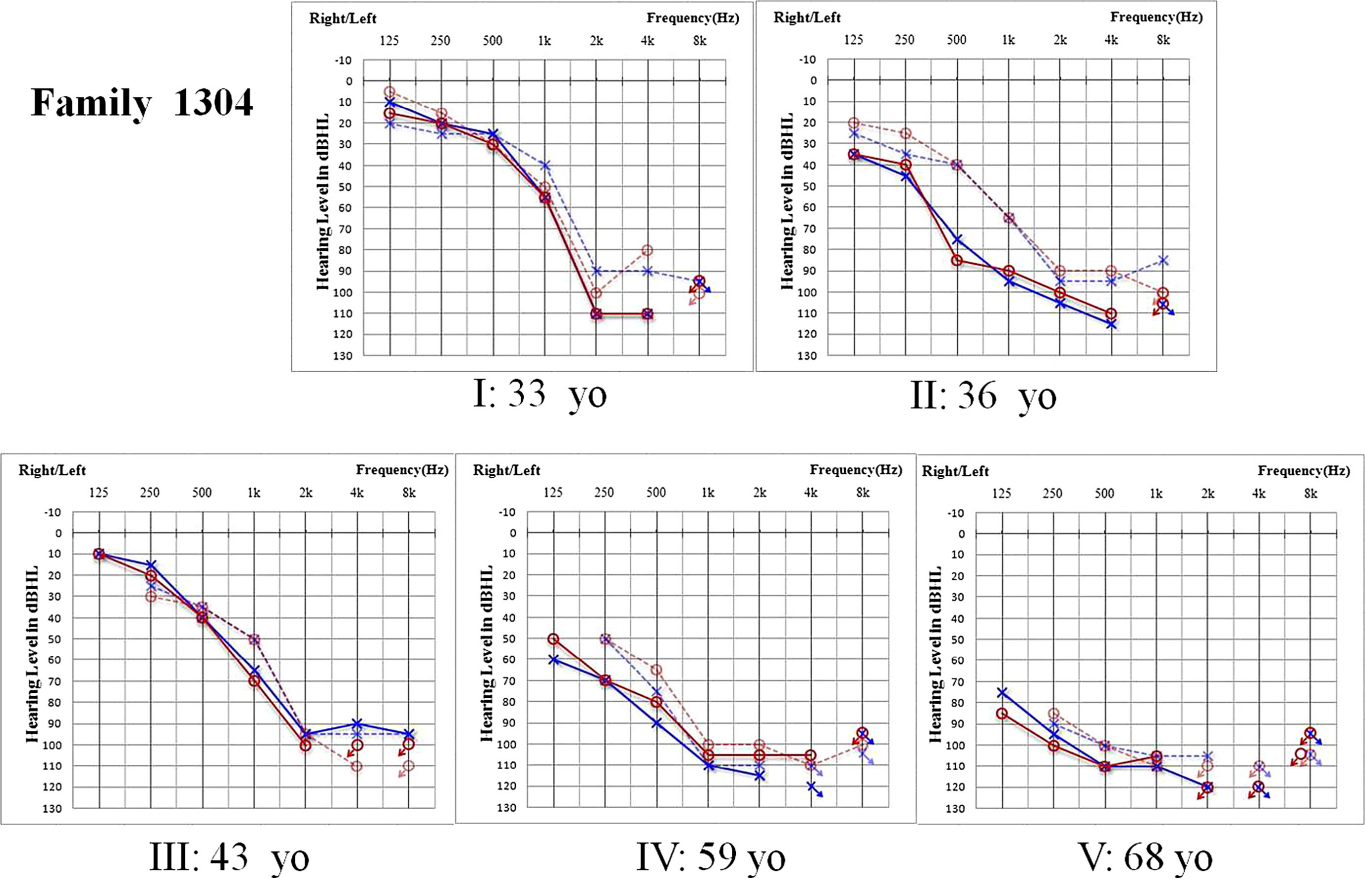
Audiograms of the five affected members from Family 1304. The age of the five patients ranged from 34 to 69 years old in 2015. The dashed line indicates results from 2009, and the solid line indicates results from 2015. Yo, years old

Family 1304 was a six‐generation pedigree with 222 members reported by our research team in 2014. The disease‐causing variant c.1253T>A (p.M418K) in *TMC1*, which is orthologous to the variant found in the *Bth* mouse, was identified as the pathogenic variant in this family (Zhao et al., 2014). In the auditory follow‐up of Family 1304, the PTA of subjects I and II declined over 12 dB HL in 6 years, 2 dB HL per year on an average, while the hearing of subjects III, VI, and V did not substantially change. A ceiling effect due to elevated thresholds resulted in a slower rate at older ages (Figure 5).

### 3.2 Variant detection and analysis

We identified the missense variant c.1253T>A (p.M418K) in *TMC1* (NM_138691.2) as the pathogenic variation in Family 01. The variant changes a methionine to lysine in the amino acid sequence. Notably, the variant was orthologous to the variant (p.M412K) reported in the *Bth* mouse, which is a well‐known hearing loss mouse model (Vreugde et al., 2002). Every patient, except II:1, carried the same variant, but other members with normal hearing did not. Patient II:1 had a history of using ototoxic drugs and showed high‐frequency hearing loss in the right ear and severe, all‐frequency hearing loss in the left ear. Therefore, we found that the variant cosegregated with the phenotype in Family 01. This is the second report of a *TMC1* p.K418K variant worldwide.

A known missense variant caused by a G>A substitution (c.1714G>A) in exon 19 of *TMC1* was detected in the proband of Family 02, resulting in an aspartic acid to asparagine change (p.D572N) in the translated sequence (Kurima, Yang, Sorber, & Griffith, 2003). Asparagine is an uncharged polar amino acid, a contrast to aspartic acid. This residue is located in the third intracellular loop region of the *TMC1* gene, which is conserved between different species. Sanger sequencing confirmed the cosegregation of c.1714G>A with all affected members in Family 02. The variation was not identified in the unaffected members in this family or the 1,700 Chinese control samples.

A novel likely disease‐causing homozygous missense variant (c.[797T>C];[797T>C]) in exon 13 was found in Family 03, which resulted in the replacement of isoleucine with threonine (p.[I266T];[=]). The isoleucine is located in the second extracellular loop region of *TMC1*, which is highly conserved. The c.[797T>C];[797T>C] variant cosegregated in the three‐person core family. The genotype frequency in the dbSNP137, HapMap, 1000Genomes and Exac databases was less than 0.001 (0.000). The variant was predicted to be disease‐causing by MutationTaster and probably damaging by PolyPhen‐2. The variants were absent in 1700 normal Chinese controls and 250 sporadic patients suffering from hearing loss. The nearby c.800G>A is a known pathogenic variant reported in India (Ganapathy et al., 2014). Based on all the above results and clinical features of the family, we concluded that c.[797T>C];[797T>C] in *TMC1* is likely responsible for the hearing loss in this family.

The variation c.2276G>A (p.R759H) in exon 24 of *TMC1* was identified as a SNP in Family 04. The single nucleotide change in arginine to histidine at nucleotide position 2276 was detected in five members, four affected individuals (III:7, III:9, IV:13, V:8) and one unaffected person, III:14, who showed normal hearing when being tested at 46 years old. In addition, the variant could not be detected in IV:7 who also showed hearing impairment (Figure S1). The histidine was the second amino acid of the C‐terminal of *TMC1*, which is not conserved between different species.

## 4 DISCUSSION

A total of 59 *TMC1* recessive variants and 4 dominant variants have been reported, including substitution, insertion, deletion, and splice‐site variants. The most common variant is c.100C>T, a nonsense variant that causes DFNB7/11 hearing loss. The Pakistani c.100C>T variant was derived from a common founder. The c.[797T>C];[797T>C] variant identified in Family 03 is a novel pathogenic recessive variant. Although *TMC1* recessive variants are very common worldwide, they are relatively rare in the Chinese deaf population, with only two homozygous and five compound heterozygous variants identified in previous studies. For the four *TMC1* dominant variants, c.1714G>A (p.D572N) and c.1714G>C (p.D572H) affect the same nucleotide, which is proposed to be a recurrent site of variants. The c.1714G>A detected in Family 02 is a known and common dominant variant, accounting for approximately 4.4% (3/68) of ADNSHL in the Chinese population (Gao et al., 2015). The c.1714G>A variant is located within a cytoplasmic loop between TM4 and TM5 that may play a critical role in forming the pore subunit of the transduction channel (Gao et al., 2015; Hilgert et al., 2008; Kurima et al., 2002; Labay, Weichert, Makishima, & Griffith, 2010). The c.1253T>A variant identified in Family 01 is another well‐known dominant *TMC1* variant, which has been reported by our group previously; this is the second report of this variant worldwide. The variant leads to replacement of methionine by lysine at residue 418 of the *TMC1* amino acid sequence, which is orthologous to the p.M412K variant in the *Bth* mouse (Vreugde et al., 2002; Zhao et al., 2014).

The auditory phenotypes of the individuals with *TMC1* homozygous recessive variants or compound heterozygous variants generally show congenital severe‐profound hearing impairment. The phenotype of the patient in Family 03 is consistent with that of previously reported DFNB7/11 families. The *TMC1* dominant variants related to hearing impairment showed late‐onset and progressive severe‐profound sensorineural hearing impairment, similar to the hearing loss of the members in Family 01 and Family 02. The follow‐up data of Family 1304, the previously reported family with a c.1253T>A variant, confirmed the progressive characteristics of the DFNA36 phenotype. There are several differences in phenotypes between the DFNA36 families in terms of age of onset and rate of progression. This difference may reflect varied effects of these substitution variants, different genetic backgrounds, or both (Nakanishi, Kurima, Kawashima, & Griffith, 2014).

*TMC1* has 24 exons transcribing a full‐length mRNA of 3201 base pairs and a protein of 760 amino acid residues, containing six transmembrane domains, a long cytoplasmic N‐terminus and a short C‐terminus. *TMC1* is expressed at the tips of the cochlear hair cell stereocilia, adjacent to the tip‐link, and maintains mechano‐electrical transduction (MET), the conversion of mechanical stimuli to electrical signals in mammalian hair cells (Kawashima et al., 2011; Kiyoto Kurima et al., 2003; Marcotti, Erven, Johnson, Steel, & Kros, 2006). The protein encoded by *TMC1* is necessary for converting mechanical stimuli to electrical signals in mammalian hair cells. For example, p.M418K in *Tmc1*, which affects pore formation of the transduction channel, has secondary effects on hair cell function and survival (Pan et al., 2013). There are several lines of evidence supporting the MET mechanism of *TMC1*: 1, *Bth* mice with the p.M412K variant showed reduced hair cell conductivity and calcium ion pass rate; 2, Similar findings were found in *Tmc1* deletion mice; 3, Transgenic mice with inserted exogenous *Tmc1* showed restoration of the mouse hair cell MET function and hearing; 4, *TMC1* is similar to the verified MET channel protein, and a recent study reported that *TMC6/8* is a zinc ion transporter and ion channel (Sirianant, Ousingsawat, Tian, Schreiber, & Kunzelmann, 2014); 5, *TMC1* can form ion channels heterogeneously (Chatzigeorgiou, Bang, Hwang, & Schafer, 2013); and 6, *TMC1* is closely related to one of the tip‐link proteins (Maeda et al., 2014).

Since the identification of p.M418K in *TMC1* in human patients, the *Bth* mouse has become an excellent animal model to study the mechanism for autosomal dominant hearing loss caused by the *TMC1* variant, as well as a promising model for development of treatments to correct *TMC1* variants and eventually restore hearing in human patients. For recessive cases, mice that carry *Tmc1* deletions are good loss‐of‐function models. By combining AAV2/1 and the chicken β‐actin promoter, Askew et al. (2015) demonstrated that exogenous *Tmc1* or *Tmc2* can restore sensory transduction and hearing of *Bth* mice and mice with the *Tmc1* deletion. By injection of Cas9:guide RNA:lipid complexes targeting the Bth allele into the cochlea of neonatal *Bth* mice, Gao et al. (2017) reduced the progressive hearing loss of *Bth* mice, providing a possible strategy to rescue auditory function of human patients with *TMC1* variants.

In summary, we identified two pathogenic variants, one likely pathogenic variant and one SNP in the *TMC1* gene by HTS in four Chinese hearing loss families, including the second report of the p.M412K variant orthologous to that in *Bth* mice, which are a good model for gene therapy analysis. In addition, we elucidated the genotype‐phenotype correlation of different *TMC1* inheritance patterns and enriched the variant spectrum of *TMC1*.

## CONFLICT OF INTEREST

The authors have declared that no competing interests exist.

## AUTHOR CONTRIBUTIONS

Conceived and designed the experiments: QW, HW, KW. Performed the experiments: HW, KW, JG. Analyzed the data: HW, KW, JG. Contributed reagents/materials/analysis tools: KW, HW, JG, JY, LX, FX, LL. Wrote the paper: HW, KW, QW Critical reading and discussion of manuscript: DW, QW.
