# Supplementary material for: Identification of four TMC1 variations in different Chinese families with hereditary hearing loss
Source: Mol Genet Genomic Med. 2018 Apr 14;6(4):504–13. doi: 10.1002/mgg3.394 (PMC6081220; doi:10.1002/mgg3.394)
Supplement: Supplementary file 1 [file MGG3-6-504-s001.docx]

**Supplementary file 1. Table 1 Overview of *TMC1* homozygous variants associated with autosomal recessive hearing loss to date**

| **NO** | **Sequence Variations** | | **Mutation forms** | **Exon/ Intron** | **Origin** | | **Hearing Impairment** | | **References** |
| --- | --- | --- | --- | --- | --- | --- | --- | --- | --- |
|  | **cDNA** | **Amino Acid** |  |  | **Nation** | **Family** | **Onset** | **Phenotype** |  |
| 1 | c.16+1G>T | Splice disruption | splice-site variant | I5 | Pakistan | 1 | Prelingual | Severe-profound | Kitajiri [2007][^1^](#_ENREF_1) |
| 2 | c.64+2T>A | Splice disruption | splice-site variant | I6 | Turkey | 2 | Congenital/Prelingual | Severe-profound | Simaci [2009][^2^](#_ENREF_2) |
| 3 | c.-195_16del | Genomic deletion | deletion | E5 | Pakistan | 1 | Prelingual | Severe-profound | Kurima [2002][^3^](#_ENREF_3) |
| 4 | c.100C>T | p.R34X | nonsense | E7 | Pakistan | 5 | Prelingual | Severe-profound | Kurima [2002][^3^](#_ENREF_3) |
|  |  |  |  |  | Pakistan | 5 | Prelingual | Severe-profound | Kitajiri [2007]^1^ |
|  |  |  |  |  | Lebanon/Jordan | 1 | Congenital | Severe-profound | Hilgert [2008][^4^](#_ENREF_4) |
|  |  |  |  |  | Iran | 1 |  |  | Hilgert [2008]^4^ |
|  |  |  |  |  | Tunisia | 3 | Congenital | Severe-profound | Tlili [2008][^5^](#_ENREF_5) |
|  |  |  |  |  | Turkey | 1 | Congenital/Prelingual | Severe-profound | Simaci [2009]^2^ |
|  |  |  |  |  | Indian | 1 | / | / | Aparna G [2014][^6^](#_ENREF_6) |
|  |  |  |  |  | Pakistan | 1 | congenital | Moderate-profound | Ayesha Imtiaz[2016][^7^](#_ENREF_7) |
| 5 | c.150delT | p.N50KfsX25 | frameshift | E7 | Iran | 1 | Congenital | profound | Yang [2010][^8^](#_ENREF_8) |
| 6 | c.237-6T>G | Splice disruption | splice-site variant | I7 | Indian | 1 | Prelingual | Severe-profound | Aparna G[2014]^6^ |
| 7 | c.776+1G>A | Splice disruption | splice-site variant | E7 | Iran | 1 | Congenital | Severe-profound | Hilgert [2008]^4^ |
| 8 | c.295delA | p.K99KfsX4 | frameshift | E8 | North America | 1 | Prelingual | Severe-profound | Kurima [2002]^3^ |
| 9 | c.362+18A>G | p.Glu122Tyrfs*10 | frameshift | I8 | Pakistan | 1 | Congenital | Severe-profound | Sobia Shafique [2014][^9^](#_ENREF_9) |
| 10 | c.453+2T>C | Splice disruption | splice-site variant | I9 | Indian | 1 | Prelingual | Severe-profound | Aparna G [2014]^6^ |
| 11 | c.536-8T>A | Splice disruption | splice-site variant | I10 | Pakistan | 1 | Prelingual | Severe-profound | Kurima [2002]^3^ |
|  |  |  |  |  | Pakistan | 1 | Prelingual | Severe-profound | Santos [2005][^10^](#_ENREF_10) |
| 12 | c.582G>A | p.W194X |  | E11 | Turkey | 1 | / | / | Duygu Duman,[2011][^11^](#_ENREF_11) |
| 13 | c.596A>T | p.N199I | missense | E11 | Pakistan | 1 | congenital | Moderate-profound | Ayesha Imtiaz[2016]^7^ |
| 14 | c.628_630delATC | p.I210del | deletion | E11 | Indian | 1 | Prelingual | Severe-profound | Aparna G [2014]^6^ |
| 15 | c.767delT | p.F255FfsX14 | deletion | E13 | Turkey | 1 | Congenital | Severe-profound | Hilgert [2008]^4^ |
| 16 | c.776A>G | p.Y259C | missense | E13 | Turkey | 1 | Prelingual | Profound | Kalay [2005][^12^](#_ENREF_12) |
| **17** | **c.797T>C** | **p.I266T** | **missense** | **E13** | **China** | **1** | **congenital** | **Profound** | **This study** |
| 18 | c.800G>A | p.G267E | missense | E13 | Indian | 1 | Prelingual | Severe-profound | Aparna G [2014]^6^ |
| 19 | c.821C>T | p.P274L | missense | E13 | Turkey | 1 | Prelingual | Profound | Kalay [2005]^12^ |
| 20 | c.830A>G | p.Y277C | missense | E13 | Pakistan | 1 | Prelingual | Severe-profound | Santos [2005]^10^ |
| 21 | c.884+1G>A | Splice disruption | splice-site variant | I13 | Pakistan | 1 | Prelingual | Severe-profound | Kurima [2002]^3^ |
| 22 | c.1080_1084delGATCA | p.R362PfsX6 | frameshift | E15 | Turkey | 1 | / | / | Guney Bademci [2016][^13^](#_ENREF_13) |
| 23 | c.1083_1087del | p.R362pfrX6 | frameshift | E15 | Turkey | 1 | Prelingual | Profound | Kalay [2005]^12^ |
| 24 | c.1114G>A | p.V372M | missense | E15 | Pakistan | 2 | Prelingual | Severe-profound | Santos [2005]^10^ |
|  |  |  |  |  | Indian | 1 | / | / | Aparna G [2014]^6^ |
| 25 | c.1165C>T | p.R389X | nonsense | E15 | Lebanon/Jordan | 1 | Congenital | Profound | Hilgert [2008]^4^ |
|  |  |  |  |  | Tunisia | 1 | Congenital | Profound | Tlili [2008][^5^](#_ENREF_5) |
| 26 | c.1166G>A | p.R389Q | missense | E15 | Turkey | 1 | Congenital | Severe-profound | Hilgert [2008]^4^ |
|  |  |  |  |  | Pakistan | 1 | congenital | Moderate-profound | Ayesha Imtiaz[2016]^7^ |
| 27 | c.1209G>C | p.W403C | missense | E 15 | China | 1 | Prelingual | Severe-profound | Yang [2013][^14^](#_ENREF_14) |
| 28 | c.1330G>A | p.G444R | missense | E16 | Turkey | 1 | Congenital/Prelingual | Severe-profound | Simaci [2009]^2^ |
| 29 | c.1333C>T | p.R445C | missense | E16 | Turkey | 1 | Congenital/Prelingual | Severe-profound | Simaci [2009]^2^ |
|  |  |  |  |  | Indian | 1 | / | / | Aparna G [2014]^6^ |
| 30 | c.1334G>A | p.R445H | missense | E16 | Turkey | 1 | Prelingual | Profound | Kalay [2005]^12^ |
|  |  |  |  |  | Pakistan | 1 | Prelingual | Profound | Santos [2005]^10^ |
| 31 | c.1404+1G>T | Splice site | deletion | I16 | Pakistan | 1 | congenital | Moderate-profound | Ayesha Imtiaz[2016]^7^ |
| 32 | c.1534C>T | p.R512X | nonsense | E17 | Pakistan | 1 | Prelingual | Severe-profound | Kurima [2002]^3^ |
|  |  |  |  |  | Turkey | 1 | / | / | Guney Bademci,[2016]^13^ |
| 33 | c.1543T>C | p.C515R | missense | E17 | Pakistan | 2 | Prelingual | Severe-profound | Kitajiri [2007]^1^ |
| 34 | c.1566+1G>A | Splice disruption | splice-site variant | I17 | Indian | 1 | Prelingual | Severe-profound | Aparna G [2014]^6^ |
| 35 | c.1589_1590delCT | p.S530X | frameshift | E18 | Iran | 1 | congenital | Profound | Michael S. Hildebrand,[2010][^15^](#_ENREF_15) |
| 36 | c.1696_2283del | Genomic deletion | deletion | 19-24 | Turkey | 1 | Congenital/Prelingual | Severe-profound | Simaci [2009]^2^ |
| 37 | c.1763+3A>G | p.W588wFSx81 | frameshift | I19 | Netherlands | 1 | Post-lingual | Profound 1st decade: high frequencies 2nd-3rd decade: all frequencies | de Heer [2011][^16^](#_ENREF_16) |
| 38 | c.1764G>A | p.W588X | nonsense | E20 | Tunisia | 1 | Congenita | Profound | Tlili [2008]^5^ |
| 39 | c.1788C>A | p.S596R | missense | E20 | Pakistan | 1 | congenital | Moderate-profound | Ayesha Imtiaz[2016]^7^ |
| 40 | c.1810C>T | p.R604X | nonsense | E20 | Greece | 1 | Congenita | Profound | Hilgert [2008]^4^ |
|  |  |  |  |  | Morocco | 1 | Prelingual | Profound | Amina Bakhchane[2015][^17^](#_ENREF_17) |
| 41 | c.1959C>G | p.Y653X | missense | E20 | Turkey | 1 | / | / | Guney Bademci [2016]^13^ |
| 42 | c.1960A>G | p.M654V | missense | E20 | North America | 1 | Prelingual | Severe-profound | Kurima [2002]^3^ |
| 43 | c.1979C>T | p.P660L | missense | E20 | China | 1 | prelingual | Profound | Jiongjiong Hu [2016][^18^](#_ENREF_18) |
| 44 | c.2004T>G | p.S668R | missense | E21 | Pakistan | 2 | Prelingual | Severe-profound | Santos [2005]^10^ |
|  |  |  |  |  | Pakistan | 1 | Prelingual | Severe-profound | Kitajiri [2007]^1^ |
| 45 | c.2030T>C | p.I677T | missense | E21 | Turkey | 1 | Congenital/Prelingual | Severe-profound | Simaci [2009]^2^ |
|  |  |  |  |  | Iran | 1 | Prelingual | Severe-profound | Davoudi-Dehaghani, E[2015][^19^](#_ENREF_19) |
| 46 | c.2035G>A | p.E679K | missense | E21 | Pakistan | 1 | Prelingual | Severe-profound | Santos [2005]^10^ |
| 47 | c.2050G>A | p.D684N | missense | E21 | Turkey | 1 | / | / | Guney Bademci [2016]^13^ |
| 48 | c.2260+2T>A | Splice disruption | splice-site variant | I23 | Tunisia | 1 | Prelingual | Severe-profound | Zied Riahi[2014][^20^](#_ENREF_20) |

**Supplementary file 1. Table 2 Overview of *TMC1* compound heterozygous variants associated with autosomal recessive hearing loss to date**

|  | **Sequence Variations** | | **Exon/ Intron** | **Origin** | | **Hearing impairment** | | **References** |
| --- | --- | --- | --- | --- | --- | --- | --- | --- |
| **NO.** | **cDNA** | **Amino Acid** |  | **Nation** | **Family** | **Onset** | **Phenotype** |  |
| 1 | c.IVS19+5G>A | Splice disruption | I19 | Sudan | / | Congenital | Profound | Meyer et al.[2005][^21^](#_ENREF_21) |
|  | c.1165C>T | p.R389X | E15 |  |  |  |  |  |
| 2 | c.1810C>T | p.R604X | E20 | Morocco | D28C | Not available | Profound | Browmstein Z[2011][^22^](#_ENREF_22) |
|  | c.1939T>C | p.S647P | E20 |  |  |  |  |  |
| 3 | c.150delT | p.N50KfsX25 | E7 | China | D419 | Congenital | Profound | Yang et al [2013][^14^](#_ENREF_14) |
|  | c.1107C>A | p.N369K | E15 |  |  |  |  |  |
| 4 | c.236+1G>C | Splice disruption | I7 | China | D472 | Prelingual | Severe-profound | Yang et al [2013]^14^ |
|  | c.1334G>A | p.R445H | E16 |  |  |  |  |  |
| 5 | c.458G>A | p.W153X | E10 | Europe | 21 | Prelingual | Moderate -profound | Schrauwen I et al [2013][^23^](#_ENREF_23) |
|  | c.1763+3A>G | p.W588wFSx81 | I19 |  |  |  |  |  |
| 6 | c.589G>A | p.G197R | E11 | China | 1953 | Congenital | Severe-profound | Gao X et al [2013][^24^](#_ENREF_24) |
|  | c.1171C>T | P.Q391X | E15 |  |  |  |  |  |
| 7 | c.1396_1398AAC | p.N466del | E16 | China | Tibetan Chinese family | Prelingual | Severe-profound | Fangzhu Lin[2014][^25^](#_ENREF_25) |
|  | c.2210_2211insCT | p.E737HfsX2 | E23 |  |  |  |  |  |
| 8 | c.1247T>G | p.L416R | E16 | China | KLX10 | Prelingual | Severe-to-profound | Ying Chen[2015][^26^](#_ENREF_26) |
|  | c.1312G>A | p.A438T | E16 |  |  |  |  |  |
| 9 | c.64+2T>A | splice | I6 | Turkey | 393 | / | / | Guney Bademci,[2016][^13^](#_ENREF_13) |
|  | c.236+1G>A | splice | I7 | Turkey |  |  |  |  |
| 10 | c.1718T>A | p.I573N | E19 | Ecuador | 1268 | / | / | Guney Bademci,[2016]^13^ |
|  | c.2130-1delG | splice | E22 |  |  |  |  |  |

**Supplementary file 1. Table 3 Overview of *TMC1* variants associated with autosomal dominant hearing loss to date**

| **Nation** | **Family** | **cDNA** | **Protein** | **Exon** | **Phenotype** | **Reference** |
| --- | --- | --- | --- | --- | --- | --- |
| North America (Caucasian) | LMG128 | c.1714G>A | p.D572N | 19 | Profound 1st decade: mid and high frequencies 2nd decade: all frequencies | Kurima [2002]^3^ |
| North America (Caucasian) | Family H | c.1714G>A | p.D572N | 19 | Severe to profound，1st decade: mid and high frequencies，Rapid progression | Hilgert [2008]^4^ |
| China | 5315  5879/8989 | c.1714G>A | p.D572N | 19 | 1st-2nd decade: high frequencies 4th–5th decade: all frequencies，severe to profound | GAO X [2015][^27^](#_ENREF_27) |
| **China** | **Family-02** | **c.1714G>A** | **p.D572N** | **19** | 2nd-3rd decade: high frequencies，4th-5th decade:severe，all frequencies. slow progression | **This Study** |
| North America (Caucasian) | LMG248 | c.1714G>C | p.D572H | 19 | 2nd-3rd decade: high frequencies，4th-5th decade:severe，all frequencies. slow progression | Kitajiri [2007]^1^ |
| Iran | L1754 | c.1249G>A | p.G417R | 16 | Severe to profound 2nd decade: all frequencies,  Rapid progression | Yang [2013][^14^](#_ENREF_14) |
| China | 1304 | c.1253T>A | p.M418K | 16 | onset age: 5~28 years old. Middle-high frequences,5th~6th decade: severe to profound, all frequencies | Zhao YL [2014][^28^](#_ENREF_28) |
| **China** | **Family-01** | **c.1253T>A** | **p.M418K** | **16** | **onset age：8-20 years old. All frequencies, moderate, 3rd decade: moderate-severe** | **This Study** |

**References**

1 Kitajiri, S. I. et al. Identities, frequencies and origins of TMC1 mutations causing DFNB7/B11 deafness in Pakistan. Clinical genetics 72, 546-550, doi:10.1111/j.1399-0004.2007.00895.x (2007).

2 Sirmaci, A. et al. Mutations in TMC1 contribute significantly to nonsyndromic autosomal recessive sensorineural hearing loss: a report of five novel mutations. International journal of pediatric otorhinolaryngology 73, 699-705, doi:10.1016/j.ijporl.2009.01.005 (2009).

3 Kurima, K. et al. Dominant and recessive deafness caused by mutations of a novel gene, TMC1, required for cochlear hair-cell function. Nature genetics 30, 277-284, doi:10.1038/ng842 (2002).

4 Hilgert, N. et al. Mutation analysis of TMC1 identifies four new mutations and suggests an additional deafness gene at loci DFNA36 and DFNB7/11. Clinical genetics 74, 223-232, doi:10.1111/j.1399-0004.2008.01053.x (2008).

5 Tlili, A. et al. TMC1 but not TMC2 is responsible for autosomal recessive nonsyndromic hearing impairment in Tunisian families. Audiology & neuro-otology 13, 213-218, doi:10.1159/000115430 (2008).

6 Ganapathy, A. et al. Non-syndromic hearing impairment in India: high allelic heterogeneity among mutations in TMPRSS3, TMC1, USHIC, CDH23 and TMIE. PloS one 9, e84773, doi:10.1371/journal.pone.0084773 (2014).

7 Imtiaz, A. et al. Recessive mutations of TMC1 associated with moderate to severe hearing loss. Neurogenetics 17, 115-123, doi:10.1007/s10048-016-0477-1 (2016).

8 Yang, T. et al. A novel mutation adjacent to the Bth mouse mutation in the TMC1 gene makes this mouse an excellent model of human deafness at the DFNA36 locus. Clinical genetics 77, 395-398, doi:10.1111/j.1399-0004.2009.01338.x (2010).

9 Shafique, S. et al. Genetic spectrum of autosomal recessive non-syndromic hearing loss in Pakistani families. PloS one 9, e100146, doi:10.1371/journal.pone.0100146 (2014).

10 Santos, R. L. et al. Novel sequence variants in the TMC1 gene in Pakistani families with autosomal recessive hearing impairment. Hum Mutat 26, 396, doi:10.1002/humu.9374 (2005).

11 Duman, D., Sirmaci, A., Cengiz, F. B., Ozdag, H. & Tekin, M. Screening of 38 genes identifies mutations in 62% of families with nonsyndromic deafness in Turkey. Genetic testing and molecular biomarkers 15, 29-33, doi:10.1089/gtmb.2010.0120 (2011).

12 Kalay, E. et al. Four novelTMC1 (DFNB7/DFNB11) mutations in Turkish patients with congenital autosomal recessive nonsyndromic hearing loss. Human Mutation 26, 591-591, doi:10.1002/humu.9384 (2005).

13 Bademci, G. et al. Comprehensive analysis via exome sequencing uncovers genetic etiology in autosomal recessive nonsyndromic deafness in a large multiethnic cohort. Genetics in medicine : official journal of the American College of Medical Genetics 18, 364-371, doi:10.1038/gim.2015.89 (2016).

14 Yang, T., Wei, X., Chai, Y., Li, L. & Wu, H. Genetic etiology study of the non-syndromic deafness in Chinese Hans by targeted next-generation sequencing. Orphanet journal of rare diseases 8, 85, doi:10.1186/1750-1172-8-85 (2013).

15 Hildebrand, M. S. et al. Mutations in TMC1 are a common cause of DFNB7/11 hearing loss in the Iranian population. The Annals of otology, rhinology, and laryngology 119, 830-835 (2010).

16 de Heer, A. M. et al. Progressive sensorineural hearing loss and normal vestibular function in a Dutch DFNB7/11 family with a novel mutation in TMC1. Audiology & neuro-otology 16, 93-105, doi:10.1159/000313282 (2011).

17 Bakhchane, A. et al. A novel mutation in the TMC1 gene causes non-syndromic hearing loss in a Moroccan family. Gene 574, 28-33, doi:10.1016/j.gene.2015.07.075 (2015).

18 Hu, J. et al. Exome sequencing identifies a mutation in TMC1 as a novel cause of autosomal recessive nonsyndromic hearing loss. Journal of translational medicine 14, 29, doi:10.1186/s12967-016-0780-5 (2016).

19 Davoudi-Dehaghani, E., Fallah, M. S., Tavakkoly-Bazzaz, J., Bagherian, H. & Zeinali, S. Allelic heterogeneity among Iranian DFNB7/11 families: report of a new Iranian deaf family with TMC1 mutation identified by next-generation sequencing. Acta oto-laryngologica 135, 125-129, doi:10.3109/00016489.2014.969383 (2015).

20 Riahi, Z. et al. Whole exome sequencing identifies new causative mutations in Tunisian families with non-syndromic deafness. PloS one 9, e99797, doi:10.1371/journal.pone.0099797 (2014).

21 Meyer, C. G. et al. Novel TMC1 structural and splice variants associated with congenital nonsyndromic deafness in a Sudanese pedigree. Hum Mutat 25, 100, doi:10.1002/humu.9302 (2005).

22 Brownstein, Z. et al. Targeted genomic capture and massively parallel sequencing to identify genes for hereditary hearing loss in Middle Eastern families. Genome biology 12, R89, doi:10.1186/gb-2011-12-9-r89 (2011).

23 Schrauwen, I. et al. A sensitive and specific diagnostic test for hearing loss using a microdroplet PCR-based approach and next generation sequencing. American journal of medical genetics. Part A 161A, 145-152, doi:10.1002/ajmg.a.35737 (2013).

24 Gao, X. et al. Novel compound heterozygous TMC1 mutations associated with autosomal recessive hearing loss in a Chinese family. PloS one 8, e63026, doi:10.1371/journal.pone.0063026 (2013).

25 Lin, F. et al. Autosomal recessive non-syndromic hearing loss is caused by novel compound heterozygous mutations in TMC1 from a Tibetan Chinese family. International journal of pediatric otorhinolaryngology 78, 2216-2221, doi:10.1016/j.ijporl.2014.10.016 (2014).

26 Chen, Y. et al. Targeted next-generation sequencing in Uyghur families with non-syndromic sensorineural hearing loss. PloS one 10, e0127879, doi:10.1371/journal.pone.0127879 (2015).

27 Gao, X. et al. Targeted gene capture and massively parallel sequencing identify TMC1 as the causative gene in a six-generation Chinese family with autosomal dominant hearing loss. American journal of medical genetics. Part A 167A, 2357-2365, doi:10.1002/ajmg.a.37206 (2015).

28 Zhao, Y. et al. A novel DFNA36 mutation in TMC1 orthologous to the Beethoven (Bth) mouse associated with autosomal dominant hearing loss in a Chinese family. PloS one 9, e97064, doi:10.1371/journal.pone.0097064 (2014).
