# Supplementary material for: Identification of four TMC1 variations in different Chinese families with hereditary hearing loss
Source: Mol Genet Genomic Med. 2018 Apr 14;6(4):504–13. doi: 10.1002/mgg3.394 (PMC6081220; doi:10.1002/mgg3.394)

Table 1. Primers sequences for four different variations in *TMC1*


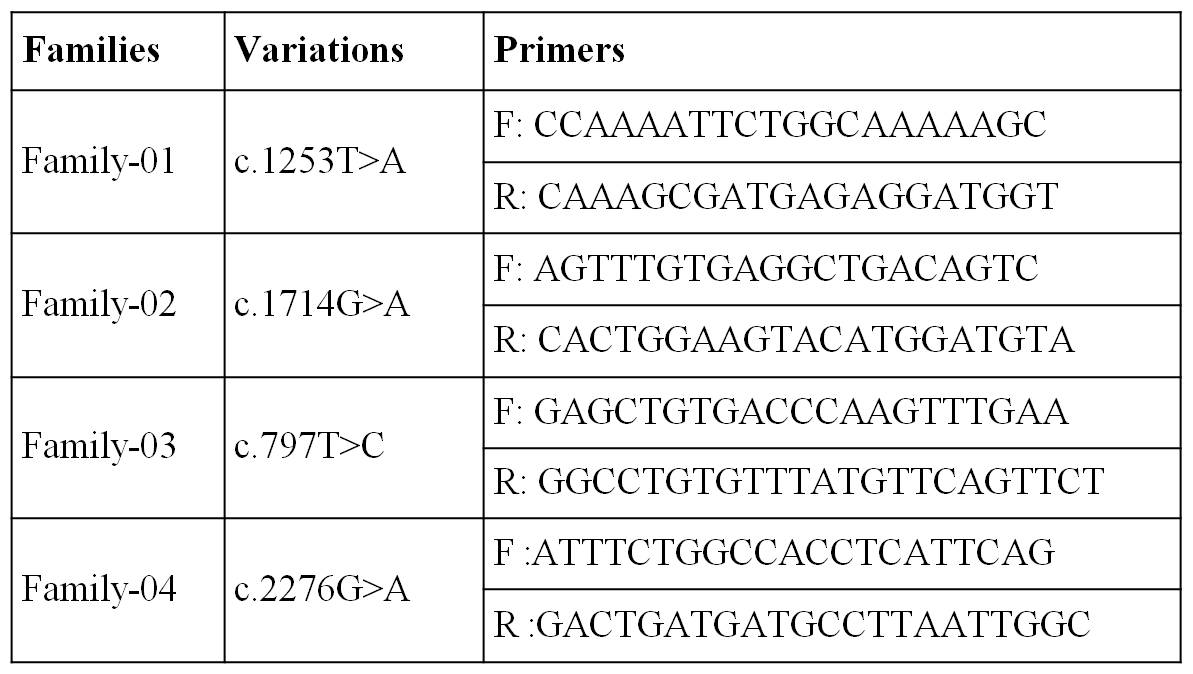


Table 2. [Reaction](javascript:void(0);) [system](javascript:void(0);) of the polymerase chain reaction (PCR)

| **Reagent** | **Volume** |
| --- | --- |
| 2X Goldstar Buffer Mix | 10ul |
| H_2_O | 7ul |
| DNA | 80ng(≈1ul) |
| Primer Mix | 2u |

Table 3. [Reaction](javascript:void(0);) [condition](javascript:void(0);) of the PCR


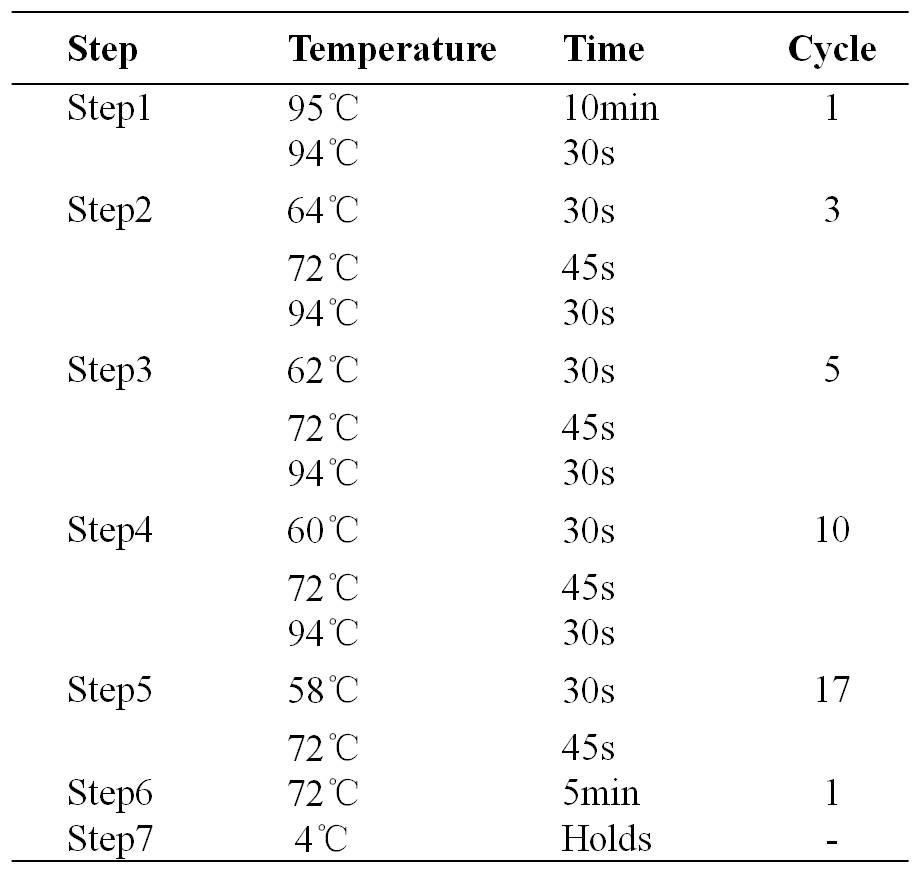

Supplement: Supplementary file 2 [file MGG3-6-504-s002.docx]
