# Supplementary figures and images for: Identification of four TMC1 variations in different Chinese families with hereditary hearing loss
Source: Mol Genet Genomic Med. 2018 Apr 14;6(4):504–13. doi: 10.1002/mgg3.394 (PMC6081220; doi:10.1002/mgg3.394)

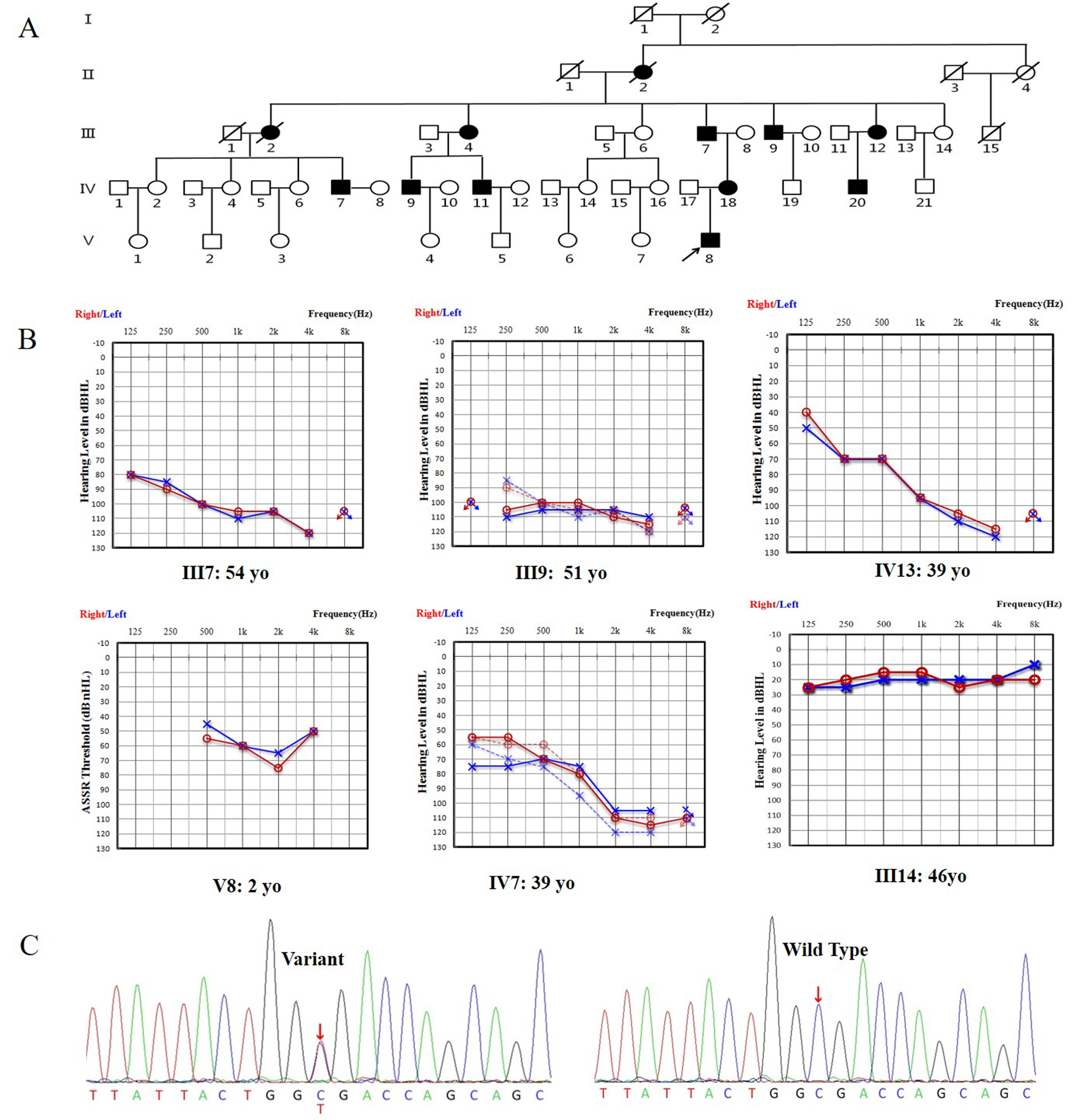

Supplement: Supplementary file 4 [file MGG3-6-504-s004.tif]
