# Supplementary material for: Identification of four TMC1 variations in different Chinese families with hereditary hearing loss
Source: Mol Genet Genomic Med. 2018 Apr 14;6(4):504–13. doi: 10.1002/mgg3.394 (PMC6081220; doi:10.1002/mgg3.394)
Supplement: Supplementary file 5 [file MGG3-6-504-s005.docx]

**Supplementary files**

**Supplementary File 1.** Overview of *TMC1* pathogenic variants identified to date. Table 1. Overview of *TMC1* homozygous variants at DFNB7/11 locus identified to date; Table 2 Overview of *TMC1* compound heterozygous variants at DFNB7/11 locus identified to date; Table 3 Overview of TMC1 variants at DFNA36 locus identified to date.

**Supplementary File 2.** Primer sequences, [reaction](javascript:void(0);) [system](javascript:void(0);) and [reaction](javascript:void(0);) [condition](javascript:void(0);)s of the polymerase chain reaction (PCR). Table 1. Primers sequences for four different variations in *TMC1*; Table 2. Reaction system of the polymerase chain reaction (PCR); Table 3. Reaction condition of the PCR.

**Supplementary File 3** List of the targeted 127 genes or related regions

**Supplementary File 4. Genotype and phenotype of Family 04.** A) Pedigree of Chinese Family 04 with ADNSHL. Affected subjects are denoted in black. The proband is indicated by an arrow. B) Audiograms of the proband and other family members. III:7, III:9, IV:13, V:8 are the family members with hearing loss and *TMC1* variant; IV:7 is the family member with hearing loss but no *TMC1* variant; III:14 is the family member with normal hearing and *TMC1* variant. C) Sequencing chromatograms (antisense sequence) of *TMC1* show the variant c.2276G>A.
